# A balance between putting on the researcher’s hat and being a fellow human being: a researcher perspective on informal carer involvement in health and social care research

**DOI:** 10.1186/s12961-022-00946-8

**Published:** 2022-12-16

**Authors:** Camilla Malm, Håkan Jönson, Stefan Andersson, Elizabeth Hanson

**Affiliations:** 1 Department of Social Work, Swedish Family Care Competence Centre, Linnaeus University, Växjö, Sweden; 2 School of Social Work, Lund University, Lund, Sweden; 3 Department of Health and Caring Sciences, Swedish Family Care Competence Centre, Linnaeus University, Kalmar, Sweden

**Keywords:** Informal carer involvement, Researchers, Health and social care research, Public and patient involvement (PPI), Interviews, Discourse psychology

## Abstract

**Background:**

Public involvement in health and social care research is increasingly prioritized by policy-makers and research funders. Often, the impact of the involvement is described in terms of how it has contributed to the research outcomes and how it has affected the involved members of the public. There is a dearth of studies reporting from the perspective of researchers themselves of having involved members of the public in their research. Nevertheless, there is a general expectation for researchers to accept and embrace public involvement in research. This study aims to explore researchers’ views of involving informal carers in health and social care research.

**Methods:**

Eleven individual in-depth interviews with researchers in the fields of social work, caring science, health science and medical science constituted the dataset of this qualitative study, inspired by discourse psychology.

**Results:**

The qualitative data analysis resulted in two interpretative repertoires describing researchers’ views of involving informal carers in research, “Philosophy of Science” and “Personal relationships and growth”. Both repertoires need to be recognized; however, as of today, the Philosophy of Science repertoire is more acknowledged, while the second repertoire describing empathy, relationships and emotions may be viewed as the researcher being “unprofessional”. Further, the findings highlighted the dual perspective of being a researcher and a carer as creating opportunities for growth on the part of the researcher, on both a professional and a personal level.

**Conclusions:**

Researchers and their research work would benefit from acknowledging, discussing and reporting both interpretative repertoires in their publications, as well as recognizing the benefit of dialectal positions, for example, having a dual perspective as both a researcher and an informal carer.

## Introduction

Public and patient involvement (PPI) in health and social care research has been advocated for several decades to improve the quality of research and lead to new ideas and innovations and to more responsive support interventions [9, 28]. PPI is often defined as research carried out with or by members of the public rather than on or to them [27]. It is increasingly prioritized and valued by policy-makers across Europe, and it is not unusual for research funders in health, social care and welfare to require researchers to involve members of the public in their research in order to receive funding [22, 26]. Different frameworks and models aiming to guide researchers when working with multistakeholders have been developed to help provide the conditions for a more comprehensive understanding, ideas for solutions and to help research bring about change in practices and policy [5]. The frameworks and models describe involvement as ranging from consultation to coproduction [2, 4, 22]. Research on involvement most often reports on research impact in terms of its contribution to research outcomes [10, 14], as well as the impact on the involved members of the public [3]. To date, there is a dearth of empirical studies reporting on new knowledge, insights and perspectives, gained by researchers themselves, regarding other possible impact that may develop from involving members of the public in their research [40, 43–45].

While there is a general expectation for researchers to accept and embrace public involvement in health and social care research, such involvement might in practice be perceived as a burden [9]. Some studies have depicted involvement of the public as time consuming, slowing down the pace of research [43, 44]. Previous research has shown that researchers’ (negative) attitudes to involving the public could lead to tokenistic involvement efforts, carried out solely to meet policy requirements [45]. Providing researchers with the appropriate circumstances and rationale for collaboration with members of the public could actually present health and social care researchers with new ways of working, for example to inform changes to the research design, planning, delivery and dissemination, as well as lead to changed values, priorities and practice [43–45]. Besides the purely practical circumstances, valuing and strengthening relationships (that is, the state of being related or connected) and reciprocal trust between researchers and contributors from outside academia have been identified as key components in enabling public involvement in research [10, 48]. The above implies that reframing the impact of involvement on purely the research itself to being more balanced, including a more systematic exploration of how involvement influences researchers themselves, such as their knowledge exchange and sharing, individual learning, awareness or behaviour, could enable a deeper understanding about how involvement works – and hence how to improve practices in the area [14, 43, 44].

The public is a broad concept, often used to capture the different types of stakeholders who may be involved in health and social care research, for example patients, service users and informal carers [27]. In this study, the specific stakeholder group involved in research by the participating researchers were informal carers. This heterogeneous group, whose common denominator is often their informal caring role [25], is of particular interest in relation to research involvement and yet there is currently a dearth of empirical studies focusing on carer involvement in research [32]. Researchers may regard members of this particular category of public involvement as speaking from two different positions: firstly, as a provider of care and advocate for a family member and/or significant other, and secondly, as a person having support needs of their own due to their caring role [1, 36]. In this regard it is also important to recognize that researchers may have current or prior first-hand knowledge of being an informal carer themselves – a position that they may or may not refer to when describing involvement of informal carers in research. Kanuha [30] described such positioning as being the insider and the outsider, where insider research refers to researchers conducting research with categories of the public of which they are also members [30]. Researchers who relate involvement to their own personal experiences may be perceived by others as failing to take an objective stance, but this experience may also be interpreted as a foundation for trust and understanding [12].

From the first author’s work in the area of carer involvement in research thus far [33, 34] it can be argued that involvement of carers in research brings an added richness and diversity of knowledge and experiences to health and social care research. Exploring and reporting about researchers’ positions and views towards carer involvement in research could help to gain a more in-depth understanding of carer involvement in health and social care research.

Therefore, the aim of this study was to explore researchers’ views of informal carer involvement in health and social care research.

## Method

This study is qualitative and inspired by discursive psychology (DP), a methodology framing discourse, language and action as socially situated [11, 47], where language is seen as a way to explore different interpretations of contexts and the world. DP has its roots in symbolic interactionism and focuses on the way people form and reform their understanding of objects and phenomena within their local contexts [7]. Scholars using the DP approach have argued that phenomena such as reasons, responsibilities, identities and roles are not fixed but rather put into action using language [24, 38]. In this study, we will make use of three analytical concepts from DP: (i) positions/positioning, (ii) interpretative repertoires and (iii) subjective positions. Positions and positioning stand for references that individuals make when constructing identities and categorizations of themselves and others [16, 24, 46]. Taking a position like “since I am a researcher, I think” could for instance signal that a person has a particular authority or responsibility. Interpretive repertoires are patterned ways of understanding different phenomena – modes of talking – that usually contain different positions [38]. The concept can be illustrated with reference to one of the interpretive repertoires that were identified in the analysis. Involvement of informal carers was placed within a context of philosophy of science reasoning, where methodological concerns relating to validity and representation were applied and evaluated by researchers. The participating researchers positioned themselves and the informal carers they had involved in their studies to justify their actions [46]. These positions are called subject positions, and they describe the roles available within a specific interpretative repertoire, simultaneously as they are part of a person’s identity construction [16, 19]. The use of concepts from DP makes it possible to move beyond the understanding of researchers and informal carers as having fixed roles, through its focus on how positions are linked to experience, knowledge and authority and how researchers shift and develop different positions when describing involvement of informal carers in research.

### Participants, recruitment procedure and data collection

The qualitative data were collected via individual interviews with 11 researchers. To mirror the broadness of disciplines within health and social care research, the inclusion criteria for participating in the study included experience of involving informal carers in research or research and development work within health and social care research, in a way that went beyond purely filling in a questionnaire or participating in an interview. Also, the carer involvement should have resulted in/was planning to result in a scientific article, report or other text by the potential participant researcher.

Initially, an information letter was sent via email by the first author (C.M.) to a wide range of health and social care researchers in Sweden. All coauthors helped to disseminate the information letter via personal contacts and networks, including a request to the recipient researchers to forward the email to other researchers they considered would be potentially interested in participating in an interview. Researchers who were interested in participating communicated this by sending a return email. No reminders were sent to those who did not answer. Step two was to send a confirmation email to those researchers who were interested in participating, including a Doodle (an online scheduling tool) link where the presumptive participants could choose a mutually agreeable time for the interview from several meeting times offered. Eleven researchers showed an interest in being interviewed, all were invited to participate and all took part.

The sample consisted of 11 researchers, 10 women and 1 man. They were aged between 36 and 65 years (*M* = 44), and all had Swedish as their first language. The participants’ educational levels varied; the majority (*n* = 9) had a doctoral degree, and the remaining two participants had master’s degrees. One participant worked as a project leader at a nongovernmental organization whilst 10 participants were employed at Swedish universities. Participant researchers reported belonging to a range of disciplines including social work, caring science, health science and medical science. Their seniority as researchers ranged from assistant professor to that of professor. All participating researchers had actively involved informal carers in recent years (past 10 years) and for longer periods of time, stretching from months to several years. Several participants had conducted more than one project involving informal carers during their research careers. The participant researchers had mainly conducted single qualitative studies, but some also described managing more extensive research programmes including both qualitative and quantitative studies. The methods used when involving carers varied, and some participants had used several different methods. The most commonly reported involvement methods were repeated interviews, focus groups, repeated questionnaires and user panels for the purposes of consultation and coproduction.

Due to the COVID-19 pandemic, all interviews were carried out online via the Zoom video conference system. A conversational interview guide based on the study’s core aims was used. The guide included the following themes: (1) personal views of carer involvement in research, (2) carers’ motivations and obstacles for research involvement, (3) carers’ knowledge contributions to research and (4) personal transformation and acquired knowledge as a result of carer involvement in research. C.M. performed all online interviews, which lasted between 60 and 75 min.

### Recordings, transcription and analysis

With participants’ permission, all interviews were fully audio recorded and thereafter transcribed verbatim. The transcriptions were continuously entered into the NVivo software (released in March 2020), and directly afterwards the analysis commenced. First, the texts were thoroughly read; thereafter, sections of text were openly coded and sections with similar content were organized into themes. Second, the researchers’ ways of talking about carer involvement in their research were explored, and patterns, agreements, contradictions and positions formed two interpretative repertoires. In these repertoires, positions tied to authority, values and responsibilities became visible. The translations of the excerpts, transcribed by C.M. from Swedish to English were scrutinized by a native English speaker and coauthor (E.H.).

### Findings

We identified two interpretative repertoires that we will refer to as a “Philosophy of Science” repertoire and “Personal relationships and growth”. The two repertoires are presented separately, despite being intertwined and belonging to the same discourse: carer involvement in research from a researcher perspective.

### Philosophy of science

This first interpretative repertoire included talk about the research process, methodological considerations and the value of carer involvement in different stages of the research process, described by the subject position of the methodologist, a position developed through methodological considerations on what knowledge carers bring to research and how to evaluate problems related to heterogeneity and representation. Central to the repertoire was that carers involved in research were described as research subjects, applying the subject position of the resource.

The research contributions of carers were described as substantial, and carers were seen as having a potential to take on a dual perspective, comprising the position of the insider and the outsider, that is, talking both as part of and from outside the relationship between a carer and a care recipient, as well as having a capacity to include the voices from other carers. The participants described the knowledge contributed by carers as emotive and situated in a relationship (between the carer and the care recipient) no longer in balance due in part to being old, ill or having a disability, as well as being complex, holistic and context bound, going well beyond pure caring experiences. The knowledge brought to research by carers was described as subjective everyday expert knowledge that researchers cannot obtain in any other way, or, as phrased by Borkman, as a privileged knowledge source [8]. In the excerpt below, the researcher mediated surprise regarding the extensive contributions of informal carers, which was far beyond what was originally anticipated by the researcher concerned:

I’m kind of surprised about how valuable the carers have been (…) it’s like a door has been opened to so much more than I thought, and this project has taken other directions after we talked to the carers (Lucy).

Besides mediating personal experiences, carers were expected to have the capability to adapt a broader perspective, as described in the following excerpt: *… to be competent in discussing outside themselves, yet including their own experience, but avoiding carers who wallow in their own problems, because that’s not the purpose, it’s not therapy-groups… (Morgan).* This excerpt shows that, even though researchers strive towards equal relationships with the participating carers, the researchers are the ones with power to define what knowledge is desirable. They were also the ones with power and the mandate to make decisions in the research relationship, for reasons phrased as based on their superior knowledge on research as well as their responsibility to report back to funding bodies. Carer knowledge was therefore considered as in need of being valued by researchers, filtered through methodological and theoretical reasoning. This position was typically expressed as a matter of evaluation: *you need to be prepared for different values to be set against each other and that you are the one with the power to do these prioritisations (Frankie)*. The evaluative position was particularly prominent in descriptions on carers as a heterogeneous category.

In light of the perceived heterogeneity of the carer population, the participants acknowledged that some carer categories based on their educational background, gender and ethnicity more commonly choose to become involved in research. Taking a methodological approach, they emphasized the importance of recruiting samples comprising a wide variety of carers to make the research more valid. It was considered a significant challenge that the carers who choose to become involved in research are often “expert carers”, that is, those with extensive experiences of caring, resourceful with previous experiences of research involvement, possibly including having/having had an occupation which has taught them to talk the talk and to understand the principles of research. Middle-class ethnic Swedish women with a university degree, described as fighters, were perceived as overrepresented in research situations. The participants argued that, although dilemmas, emotions and a sense of guilt in reality may be similar experiences existing among different carer categories, they acknowledged that different categories within the carer population could perceive that significant differences nevertheless exist:

.. yeah, you’re sitting there as a Somali woman and think that, well there’s another Swedish well-off woman with pearl earrings at home in their nice living room, talking about how difficult it is to… yeah, you can’t relate to that, even if it might be the same emotions (Lucy).

Failing to recruit a heterogeneous sample included the risk that some carer categories make themselves spokespersons for the entire carer population; *… they’re generalizing from their own experience (…) and then that’s the case for everyone… (Morgan).* This was expressed as easily resulting in the circumstances, goals and needs of carers who do not belong to this particular group to be missed. Or, as seen in this quotation: *… it’s such a shame that we gain so limited knowledge when we… are making quite a big thing of user- and carer involvement, but when you look at what you’ve done more carefully it’s been a very limited consultative effort (Ursula).* The statement could be interpreted as strongly critical of an overembellished carer involvement activity in research. In other words, such involvement could in fact be seen as tokenistic [28]. It was expressed as urgent to make considerably more efforts to reach out to and include categories of carers whose voices are seldom heard, for example, male carers, persons with a first language other than Swedish, those who experience psychological barriers to participation or those experiencing practical obstacles, such as lack of time or poor economy. To realize this, more strategic and creative recruitment was deemed necessary by the participant researchers.

The evaluative position of the repertoire was also developed in comments on when and how much carers should be involved in the research process. Some described involving carers already from the project proposal planning stage, while others involved carers recurrently in several parts of the research process, and yet others educated carers about how to contribute to panel discussions. Most frequently, however, carers were involved in recurring interviews or as collaborators in group settings. Few researchers had involved carers in the data gathering process, although they acknowledged the advantages of matching the interviewer with the interviewee (that is, carers interviewing carers), hence avoiding reduced knowledge and decreased trustworthiness in the data due to the sociodemographic belonging of the researcher. Involving carers in data gathering was also believed to include ethical issues, for example, achieving sufficient quality of the material or handling the issue of confidentiality. Even fewer carers were involved in the analysis or the writing process, since this was found to be complicated, and ideals about equally shared power were dismissed with reference to the lack of scientific competence among non-academics:

You need a certain competence to manage, you need to understand what analysis work aims to do and (…) I’ve pondered quite a lot in terms of power and involvement and the participation ladder of Arnstein and I’d say that I believe it’s very hard to bring about true collaboration where you sort of have an equivalent definition of power when you for instance interpret data (Ursula).

Incorporating carers consultatively in the analysis process was described as more realistic, for instance through discussing results or giving feedback on interventions developed. Participant carers were also commonly involved in the dissemination process, for instance by presenting results together at conferences. Nevertheless, carer involvement in all steps of the research was not seen as a goal in itself, although recognized as an appealing idea. There were multiple reasons for taking this position, for example that involvement could be time consuming and demanding a lot from the researcher in terms of flexibility and creativity, but also a recognition that demanding too extensive involvement in fact could be excluding, since some carers may lack the sufficient time and/or energy for more extensive participation. Instead, the most important aspect for successfully involving carers in research was acknowledged to be able to find a mutually meaningful level of involvement, in line with previous research [5, 37]. The frame and character of the involvement was spoken of as needing to be made explicit and accepted from all involved, already from the outset of the study/project in question. The participants’ implied clear roles, honesty and transparency were recognized as a way to decrease the risk of disagreement and of disappointment arising from a mismatch of expectations on the part of the involved carer(s) and researcher(s).

### Personal relationships and growth

This second repertoire was constructed through autobiographical accounts and mutual metaphors, considering aspects of carer involvement that go beyond pure scientific or practical aspects of research. The position of being a fellow human being and, where applicable, a carer was acknowledged and accepted, and the repertoire also involved the creation and maintenance of relationships, described through the subject position of the explorer. The corresponding position for the carer participants was that of the peer. Central to the repertoire was the concept of time and development of relationships and growth that appeared to be at odds with established values and practices of research.

The involvement was, from the researchers’ point of view, by several participants portrayed as a journey and a life-changing experience, comprising engagement and learning, but also with an emotional dimension. This journey was perceived as transcending the professional researcher role as a researcher into something new. One researcher described this in terms of *new worlds being opened*, a journey of being inspired, motivated and provided with insights and new ways of thinking. Participants explained a separation of the professional researcher role from that of the researcher’s position as a human being as impossible, since changes in views and values due to involving and meeting carers affected them both as private persons and as researchers, in a holistic way. One researcher described it as *a balance between putting on the researcher’s hat and being a fellow human being* (Sam). Another participant researcher phrased the emotional impact that provided a pathological position to researchers who were not emotionally affected when involving carers:

.. I think that the only thing that could hinder you from being affected is if you have some kind of empathy deficiency (…) the narratives are so powerful that they do something with you (…) it triggers some kind of emotion that makes you feel that… this is a difficult situation. And I think that every time you empathize with someone something happens within you (Lucy).

The researchers described feeling humble and grateful for being entrusted with carers’ stories, stories described as mediating sensitive and sometimes painful narratives of the past, of sorrow and feelings of guilt and shame as well as of injustices and of carers fighting for their rights. These narrations were explained as emotionally touching, leaving the researchers feeling sad or angry, awakening their fighting spirit, a responsibility to contribute to change and an urge to use themselves as a megaphone to spread their research and the carers’ voices. Nevertheless, the researchers emphasized that in their view the personal and emotional impact did not entail them becoming unprofessional or unscientific.

The participating researchers described having involved carers in their research for longer periods of time, sometimes in close relationships lasting for several years. Participants described an awareness about how important they, as persons, could become for those carers involved in their research, acknowledging it as a potential challenge, for instance the risk of winding up in the (one-sided) role of a potential therapist.

Some researchers described that they had discussed the duration and nature of the relationship together with participant carers already from the beginning of the project, hence avoiding expectations of forever-lasting relationships or friendships. Others wished to continue the relationship and instead talked about how they encountered external expectations of ending the relationships after the research had finished, which they found straining. The latter researchers described that, for them, involvement includes the building of relationships one cannot simply break when the carers’ input to the research is no longer needed. Instead, they emphasized a responsibility to keep an openness to continue the relationship (however transformed) after the study had ended, due to the reciprocity of the relationship and the trust and knowledge created, for example through SMS/email conversations, telephone calls or sending postcards at holiday times. The choice to continue relationships with participant carers was described as an ethical challenge not always easy to admit to, either when discussing the topic with other researchers or sometimes even to oneself.

All participating researchers described their rationale for involving informal carers in their research as emanating from having an interest in the target group; however, some acknowledged currently being in or having earlier been in a carer role themselves, possibly fuelling their engagement for the target group as well as using it strategically in their research:

… I don’t come exclusively from the worlds of research or profession you know, I have my own carer experience, which I could benefit from using there (in a focus group with carers, CM add), that is, to in some way make myself more accessible, I think (Erin).

Participants who identified as both carers and researchers talked about having a dual perspective. One researcher described their own carer experiences as creating a mutual platform together with the participating carers, while another researcher described the experiences as her carer glasses, which she could choose to wear or not to wear depending on the situation. The participants also talked about how they, during their researcher journey, had started to dare to embrace and use the personal carer identity when in the researcher role, which was viewed as contributing to becoming more courageous and to open up as a researcher when interacting with carer participants. In addition to this, some had started to reflect in a different way on their personal caring situation and gained a new understanding of their role. Personal caring experiences were believed to increase the researcher’s understanding about the context of carers, thereby contributing to the conditions for conducting research of better quality. Hence, embracing a carer identity was not described as contrary to the researcher identity, instead these different identities were believed to be able to be mergeable and advantageous to the quality of the research:

Yeah, and I think… I suppose that this is what I start to become aware of, that it’s okay to do the personal (carer) journey and the researcher journey almost in parallel. I haven’t really seen it in that way before and I think it’s really cool and I feel that this gives my research so much more (Hayden).

This repertoire goes beyond the limits of science, and the carers were seen as more than contributors to the research; they contributed to the researchers’ development as both researchers and as persons. Emotions were acknowledged and accepted, and sometimes perceived as unexpected, consistent with Sampson et al., who acknowledged that research contexts not expected to be of an emotional kind surprisingly enough may entail empathic pain (p. 927) [42]. However, the researchers were aware of and accepted the emotional impact, and described several coping strategies to handle it, such as discussions and dialogue with a colleague or a mentor. The researchers embraced their own personal emotions to different extents, but all simultaneously reported keeping their researcher identity. For some researchers, their emotions were seen as essential for doing qualitative research, and emotions like those of empathy were consciously used in their studies.

## Discussion

Two main interpretative repertoires (IRs) were identified from the data analysis: “Philosophy of Science” and “Personal relationships and growth”. We interpreted these IRs as complementary yet recognized them as possibly dichotomous, with opportunities for being competing or contradictory, which could result in ideological dilemmas [19], that is, arguing for and justifying a certain position in relation to the opposed. Despite the preconditions for both IRs to be potentially positive, we agree with Billig, who discussed the risk in pleading exclusively for one position [6]. Viewing the two repertoires as opposite poles of a continuum where the researchers, depending on personal and organizational circumstances, position themselves as well as the setup of the particular study could be a strategy to avoid dichotomous standpoints. Researchers constitute a heterogeneous group with differing views about carer involvement and how it should best be handled, originating from their personal and research positionalities (see for example [31]). Due to this, they are situated differently on the research continuum, closer or further away from either pole. This was visible in the second IR when discussing their own emotional impact, or the relationship with the involved carers, where some participants ended their relationships with the carers when the project officially ended, while others were open for a continued contact and dialogue.

A further dimension emanating from the findings and feasible to visualize on a continuum was the perspectives of the insider and the outsider [30]. In this study being an insider was most visible when researchers assumed the position of an informal carer. However, the second IR highlighted that participating researchers who were carers themselves applied a conscious, nondichotomous perspective, clearly phrased by one researcher as the “carer glasses” that could be worn or not, depending on the situation. Such a dialectical perspective was considered by participants to add further dimensions and quality to the research as well as contribute to a journey of personal development as carers and as persons, presuming an awareness about possible biases. Dwyer and Buckle [15] explained this dialectical position as the space between, challenging the dichotomy of insider versus outsider status and instead allowing the simultaneous position of both insider and outsider. We argue that the participants position themselves in ways that could be placed on a continuum, as nearer to either pole of the continuums. Their perspectives are shaped by different positions/identities, making it impossible to exclusively occupy one position. If taking the example of being a carer, a possible benefit for researchers who are also carers could be being perceived as more credible from the participating carers’ point of view, which could lead to deeper and more honest stories being told. At the same time, as will be discussed in the next section, a possible cost could be being perceived as less scientific from the research community’s point of view.

### Defending the second IR position

The distanced and evaluative position that we have identified as a core feature of the first IR has partially been challenged by proponents of feminist standpoint theory [23]. This alternative position is, however, rather challenging to apply in the case of carers, given the heterogeneous and complex character of their role. None of our interviewees referred to standpoint theory as a way of merging repertoires. The analysis revealed that the logic of the first repertoire in relation to research and researchers may be at odds with some aspects found important within the second IR. Even though the second IR, from the perspective of the participants, was viewed as contributing to the research quality, it was a position in need of being defended as not being parallel with unprofessionalism or being unscientific. In addition, the logic of the first repertoire governs the self-presentations of researchers; a curriculum vitae is unlikely to include comments on personal growth and long-standing friendships with people who have been involved in the research.

According to the second IR, participants handled the development of personal relations differently, where, as outlined above, some had negotiated the duration and content in advance while others did not find it necessary or even desirable to end the relation with the participating carers. Although not explored in this study, this dichotomy may likely depend on the researcher’s research positionality, including viewing participating carers as either the subject position the resource or the peer. The second IR highlighted a mutual influence between personhood and research, where involving carers entailed emotional responses, affecting the participants personally and professionally, making the opportunities to withdraw as a distant researcher limited. This is a finding in line with previous research [15, 41] which has shown that, when persons from outside academia are involved in a research process, the interactions between the researcher and those involved often contribute to a relational and situational research, which may result in previously unforeseen ethical dilemmas arising (see for example [50]).

Qualitative researchers are commonly expected to engage in a process of reflexivity where the personal biases, evolving from cultural, historical and geographical contexts, are revealed, aspects that from an holistic perspective should be included in the final publication [21, 39]. Our findings, indicating the first IR as the main process described in end products (for example, written reports, scientific publications), implied that researchers’ experiences of personal, relational and emotive investments traditionally are systematically excluded from being discussed and reported on in research contexts [13, 20, 21, 35]. The reasons for this may be that emotions and personal investment within the western philosophical tradition are usually considered to be subversive of knowledge, reason rather than emotion (p. 151) and the imperative for acquiring knowledge [29]. However, it has been argued that emotions and relationships both exist as well as affect the research process even if they are not reported [13, 18]. Further, engaging in their own emotions as well as those of the participants has been acknowledged in previous research as a precondition for sound qualitative research [9, 21]. We consider that accepting the possibility of personal investment and potentially being emotionally affected as a researcher, could be seen as an “insurance” for researchers to avoid becoming overwhelmed. Being overwhelmed could be a risk particularly imminent for new researchers or for those researchers who conduct emotionally difficult research, where accepting the second repertoire could contribute with an openness to talk and discuss such aspects of research with both colleagues and others who are involved in the research.

### Involvement and knowledge contributions

When stressing methodological aspects, involvement of carers according to the first IR concerned specified phases of a defined research project. The first IR highlighted that participants did not perceive carer involvement in all stages of the research process as the most important feature of the research process. Rationales for a more limited involvement varied, from an idea about how too extensive an involvement could hinder some carers from participating to acknowledging involvement in research as time consuming and demanding a lot from the researcher in terms of flexibility and creativity. Instead, transparency and having a plan for the involvement during all stages was emphasized, in line with Bammer’s 2S SEOF framework, with its toning down of the urge for stakeholders to participate in each and every part of the process. Instead, the level and realization of involvement should be grounded in the particular study concerned [4, 5]. These findings also correspond with previous research, which pointed out the core ingredients as being that of honesty and a commitment to accurately and adequately represent the brought experiential knowledge of participants [15].

However, despite the sensible justification that transparency is more important than striving for an as extensive involvement as possible, this approach includes a risk that asymmetrical relationships between researchers and carers are reinforced, and of decisions about the level of involvement being made for the wrong reasons. For instance, due to lack of time and resources, researchers may be tempted to “explain” excluding carers from certain parts of the research for reasons which are not entirely honest. This may lead the research to becoming no longer authentic, or even becoming tokenistic, possibly resulting in epistemic injustice, that is, when individuals are discriminated as knowers based on prejudices such as gender, social background or, in a broader sense, their identity [17].

### Strengths and limitations of the study

The inclusion criterion of substantive experiences of carer involvement, together with the recruitment method demanding an active response from the researcher, is mainly to be considered as a strength. Nevertheless, our sample of researchers exclusively expressed positive views of carer involvement and could thus be viewed as a narrow sample, and a limitation of the study. For example, it may have left other perspectives and challenges uncovered. We also consider our analytic method as a strength as it provided a perspective not solely describing researchers’ experiences but focusing on their views and how they talk about involving carers in their research.

## Conclusion

In this study, we explored researchers’ views of involving informal carers actively in research. We found that involving carers is complex and may include both benefits and challenges, with regard to recruitment and the research process. Carers were seen to be able to largely contribute when involved in research, not least through their dual perspective as being able to take an insider as well as an outsider perspective, that is, being those providing care at the same time as they may have support needs of their own. We established two interpretative repertoires describing researchers’ views and positions regarding involving informal carers in research, Philosophy of Science and Personal relationships and growth. Currently, the first repertoire is more acknowledged, while “admitting” the second repertoire, despite the latter being recognized as valuable and perhaps even a precondition for qualitative research, was feared to be viewed as unprofessional and unscientific.

Taking a nondichotomous position between interpretative repertoires may improve the quality of research, by influencing researchers’ individual knowledge, raising awareness about possible biases and helping to balance decisions. Researchers and their research work would benefit from acknowledging, discussing and reporting both interpretative repertoires in their publications, as well as recognizing the benefit of dialectal positions, for example, their researcher positionality or having a dual perspective as both a researcher and an informal carer. Even if this study focuses on researchers who have involved carers in their research, we believe that the findings may be relevant for researchers who involve other categories of the public as well.

## Data Availability

In keeping with the advisory opinions of the Ethical Advisory Board in Southeast Sweden, the interview data are not available beyond the core study team. This is due to the risk of identifying individual participants within the raw and analysed qualitative interview data.

## Abbreviation

IR: Interpretative repertoire
